# Toxic epidermal necrolysis in a preschooler associated with adenovirus respiratory infection: A case report

**DOI:** 10.1016/j.jdcr.2026.01.030

**Published:** 2026-01-30

**Authors:** Maria Julia Rendon-Salazar, Jimena Prieto-Gomez, Tamara Hernandez-Hernandez, Ricardo Martinez-Tenopala, Veronica Bazan-Onieva, Dalia Ibarra-Morales, Zaira Selene Mojica-Gonzale, Victor Gonzalez-Uribe

**Affiliations:** a AlergiaMx — Allergy and Clinical Immunology Unit, Mexico City, Mexico; b Facultad Mexicana de Medicina, Universidad La Salle, Mexico City, Mexico; c Hospital Infantil de México Federico Gómez — Service of Allergy and Clinical Immunology, Mexico City, Mexico; d Department of Pathology and Immunochemistry, Hospital General de Mexico, Dr Eduardo Liceaga, Mexico City, Mexico

**Keywords:** adenovirus, case report, pediatrics, Stevens-Johnson syndrome, toxic epidermal necrolysis

## Introduction

Toxic epidermal necrolysis (TEN) is a rare, life threatening mucocutaneous disorder characterized by widespread epidermal necrosis and detachment, typically classified as a type IVc hypersensitivity reaction mediated by cytotoxic T lymphocytes.^1^^,^^2^ TEN represents the most severe form within the Stevens–Johnson syndrome (SJS)/TEN spectrum. The classification is based on the extent of epidermal detachment where SJS involves less than 10% of the body surface area, SJS/TEN overlap 10% to 30%, and TEN exceeds 30%.^1^^,^^2^

The estimated annual incidence of SJS in the general population ranges from 1 to 6 cases per million people, while TEN occurs in 0.4 to 1.2 cases per million per year.^3^ In children, the annual incidence is 5.3 cases for SJS, 0.8 cases of SJS–TEN overlap, and 0.4 cases of TEN per million children.^4^

In children, the clinical presentation is challenging due to nonspecific prodromal symptoms, often resembling upper respiratory tract infections, and a wide differential diagnosis including viral exanthems, Kawasaki disease, and generalized pustulosis.^5^

Although medications are the most common triggers of TEN, particularly in adults, infectious agents play a more significant role in children.^2^^,^^5^^,^^6^ A wide range of bacteria, viruses, fungi, and parasites have been implicated as potential causes (Table I). Among these, *Mycoplasma pneumoniae is* the most frequently reported.^1^^,^^6^^,^^7^

**Table I:** Reported infectious triggers of toxic epidermal necrolysis and Stevens–Johnson syndrome

Bacterial	Viral	Fungi	Parasite
Mycoplasma pneumoniae	Enterovirus adenovirus	Coccidiomycosis histoplasmosis	Strongyloides
Yersinia	Measles		
Tuberculosis	Mumps		
Syphilis	CMV		
Chlamydia	VZV		
Streptococcus pneumoniae	HHV-6		
Salmonella	HHV-7		
Enterobacter	Influenza		
Pneumococcus	HIV		
	Hepatitis A		

*CMV*, Cytomegalovirus; *HHV*, human herpes virus; *HIV*, human immunodeficiency virus; *VZV*, varicella-zoster virus.

We report a rare case of TEN in a healthy preschool-aged child caused by polymerase chain reaction confirmed adenovirus in the absence of prior drug exposure.

## Case report

A previously healthy preschool-aged male with no recent history of immunization or medication use presented with 1 week of intermittent fever, green mucopurulent ocular discharge, rhinorrhea, and a dry cough. Forty-eight hours after symptom onset, an erythematous papular rash appeared, initially localized to the face and subsequently generalized. Clarithromycin and ampicillin were prescribed for suspected scarlet fever. However, the rash progressed rapidly the next day to painful blistering lesions and epidermal detachment.

**Fig 1:**
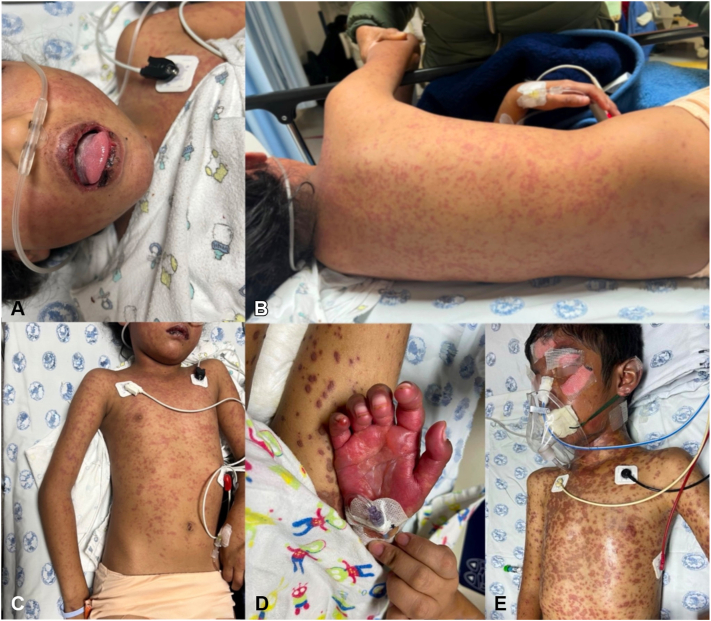
Clinical presentation of toxic epidermal necrolysis in a preschool-aged patient. **A,** Erosions of the lips and perioral mucosa; **B,** diffuse maculopapular exanthem involving the back; **C,** generalized erythematous rash with early epidermal detachment on the upper limbs; **D,** severe involvement of the palm with denuded skin, and violaceous patches; **E,** widespread *dusky* erythematous lesions with epidermal detachment over the face, chest, and arms.

He was transferred to a tertiary care center where 68% body surface area, positive Nikolsky sign, and mucosal involvement confirmed TEN. (Fig 1).

**Fig 2:**
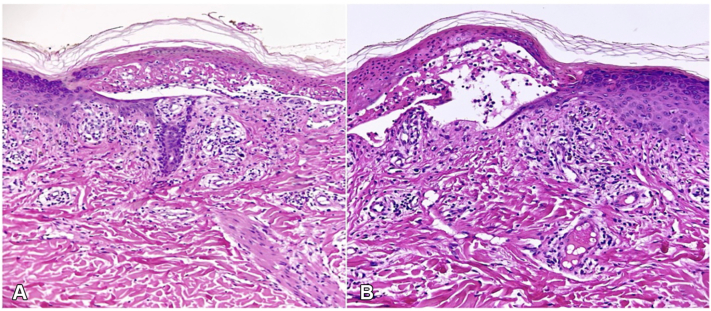
Histopathologic findings from a lesional skin biopsy. **A,** Subepidermal cleft formation with extensive keratinocyte necrosis. **B,** Basal layer vacuolization and a sparse superficial perivascular lymphocytic infiltrate (H&E, original magnification × 100).

A skin biopsy was performed from the edge of an early blister to support diagnostic confirmation. Histopathology results showed extensive keratinocyte necrosis with subepidermal cleft formation, basal layer vacuolization, and a sparse superficial perivascular lymphocytic infiltrate, findings consistent with TEN (Fig 2).

An infectious workup was conducted. Respiratory polymerase chain reaction panel was positive for adenovirus, and negative for bocavirus, coronaviruses, rhinovirus/enterovirus, influenza, metapneumovirus, parainfluenza, respiratory syncytial virus, mycoplasma pneumoniae, chlamydia pneumoniae, Legionella pneumoniae, and SARS-CoV-2. Viral load testing was negative for Epstein–Barr virus, cytomegalovirus, human herpesvirus types 6 and 7, and Kaposi's sarcoma-associated herpesvirus. Blood and stool cultures were negative. These findings confirmed adenovirus as the sole active infectious agent at the time of presentation.

The patient was managed with intravenous immunoglobulin, cyclosporine, and advanced supportive care, including biological dressings and mesenchymal cell-derived exosome therapy. His Score of Toxic Epidermal Necrolysis score was 1, indicating a relatively low predicted mortality risk. He remained hemodynamically stable without ventilatory support, and laboratory studies showed no hepatic or renal dysfunction.

The patient showed progressive re-epithelialization with no recurrence during follow-up. Persistent complications included bilateral cicatricial conjunctivitis and a right corneal ulcer requiring ophthalmologic monitoring.

## Discussion

TEN is a severe mucocutaneous condition mainly associated with drug exposure in adults; however, pediatric cases more frequently involve infectious triggers. Among these, viral infections are widely recognized, nonetheless, *Mycoplasma pneumoniae* is the most common reported trigger.^1^^,^^6^^,^^7^ Even though adenovirus is a common cause of respiratory and ocular infection in children, its association with TEN or SJS remains extremely rare.

Reports of adenovirus-associated mucocutaneous disease in children have almost exclusively described reactive infectious mucocutaneous eruption (RIME) or SJS, conditions characterized by limited or absent epidermal detachment.^7^^,^^8^ To date, published pediatric cases have not included presentations fulfilling full TEN criteria with >30% body surface area involvement.The extensive epidermal necrolysis in our patient represents a more severe phenotype within this spectrum. Table II summarizes the pediatric adenovirus-associated cases reported in the literature and illustrates how the current case extends the recognized clinical range of disease severity.

**Table II:** Published pediatric cases of adenovirus-associated mucocutaneous syndromes

Author, year	Diagnosis	BSA detachment	Adenovirus confirmation	Prior drug exposure	Outcome	Relevance to current case
Gámez-González et al, 2021	RIME	<5%	PCR positive	None	Full recovery	Mild mucositis-predominant; no epidermal necrolysis
Mentesidou et al, 2024	SJS	<10%	PCR positive	None	Full recovery	SJS without extensive skin detachment
Current case	TEN	68%	PCR positive	Macrolides given after rash onset	Ocular sequelae, no recurrence	More severe presentation; extends clinical spectrum

*BSA*, Body surface area; *PCR*, polymerase chain reaction; *RIME*, reactive infectious mucocutaneous eruption; *SJS*, Stevens–Johnson syndrome; *TEN,* toxic epidermal necrolysis.

RIME is characterized by prominent mucositis with minimal or absent skin involvement and typically lacks the widespread epidermal necrolysis observed in SJS/TEN.^7^ Although adenovirus has been reported as a trigger for RIME, the clinical presentation in our patient clearly exceeded this category. Extensive dusky erythema, blistering, positive Nikolsky sign, and >30% BSA epidermal detachment fulfills established criteria for TEN rather than RIME. This distinction is clinically important, as misclassification may underestimate disease severity and delay appropriate management.

Viral-associated mucocutaneous syndromes, particularly RIME and mild SJS, often have a more favorable course compared with classic drug-induced SJS/TEN.^5^^,^^6^ However, this pattern does not necessarily apply to TEN. In our patient, the extent of necrolysis, significant ocular sequelae, and the need for immunomodulatory therapy demonstrate that infection-triggered TEN can be as severe as drug-induced disease. Thus, the overall prognosis is determined primarily by the degree of epidermal detachment rather than by the underlying etiology.

Although the patient received clarithromycin and ampicillin due to an initial suspicion of scarlet fever, both antibiotics were administered after the onset of the rash. Moreover, these antibiotics are not among the most frequently implicated in SJS/TEN, which are more commonly associated with sulfonamides, cephalosporins, or antiepileptics.^9^ Additionally, the rash progressed rapidly to blistering and detachment within 24 hours of starting the antibiotics, which is inconsistent with the typical latency period of 4 to 28 days seen in drug-induced SJS/TEN.^9^ This temporal sequence and drug profile further support adenovirus as the primary etiologic agent in this case.

Nonetheless, further research is needed to determine whether this trend is consistent across broader populations. Clinicians should remain vigilant, as even infection-associated cases can lead to significant complications, including ocular damage and long-term cutaneous sequelae.

## Conflicts of interest

None disclosed.
